# Supplementary material for: Phage display-mediated immuno-PCR to detect low-abundance secreted proteins in Drosophila
Source: bioRxiv. 2025 Oct 31:2025.10.31.685841. Preprint. [Version 1] doi: 10.1101/2025.10.31.685841 (PMC12636453; doi:10.1101/2025.10.31.685841)
Supplement: Supplement 1 [file NIHPP2025.10.31.685841v1-supplement-1.pdf]

bioRxiv preprint doi: https://doi.org/10.1101/2025.10.31.685841; this version posted October 31, 2025. The copyright holder for this preprint (which was not certified by peer review) is the author/funder, who has granted bioRxiv a license to display the preprint in perpetuity. It is made available under aCC-BY 4.0 International license.

Supplementary figure 1. ImpL2 nanobody affinity maturation.

(A) Immunoprecipitation of ImpL2 from S2R+ cell conditioned medium using ImpL2 nanobodies. NbVHH05 was used as a negative control. The ImpL2 was detected using endogenous ImpL2 antibody.

(B) ELISA results of monoclonal phages isolated after two rounds of mutagenesis and selection of NbImpL2-1C. 96 individual clones were tested against the negative control protein (Mip-hIgG) and the target protein (ImpL2-hIgG). Clone 1A, 1B, 7A, 7B are phages displaying the original NbImpL2-1C nanobody.

(C) Sequence alignment of NbImpL2-1C variants showing improved ELISA signals in (B). Mutations in the variants are indicated. The CDR regions are outlined with red rectangles.

(D) ELISA results of monoclonal phages isolated after two rounds of mutagenesis and selection of NbImpL2-2C. 96 individual clones were tested against the negative control protein (Mip-hIgG) and the target protein (ImpL2-hIgG). Clone 1A, 1B, 7A, 7B are phages displaying the original NbImpL2-2C nanobody.

(E) Sequence alignment of NbImpL2-2C variants showing improved ELISA signals in (D). Mutations in the variants are indicated. The CDR regions are outlined with red rectangles.

(F) ELISA results of monoclonal phages isolated after two rounds of mutagenesis and selection of NbImpL2-2G. 96 individual clones were tested against the negative control protein (Mip-hIgG) and the target protein (ImpL2-hIgG). Clone 1A, 1B, 7A, 7B are phages displaying the original NbImpL2-2G nanobody.

bioRxiv preprint doi: https://doi.org/10.1101/2025.10.31.685841; this version posted October 31, 2025. The copyright holder for this preprint (which was not certified by peer review) is the author/funder, who has granted bioRxiv a license to display the preprint in perpetuity. It is made available under aCC-BY 4.0 International license.

(G) Sequence alignment of NbImpL2-2G variants showing improved ELISA signals in (F). Mutations in the variants are indicated. The CDR regions are outlined with red rectangles.

bioRxiv preprint doi: https://doi.org/10.1101/2025.10.31.685841; this version posted October 31, 2025. The copyright holder for this preprint (which was not certified by peer review) is the author/funder, who has granted bioRxiv a license to display the preprint in perpetuity. It is made available under aCC-BY 4.0 International license.

Supplementary figure 2. Quantification of ImpL2 in the hemolymph of flies bearing *Yki*-induced gut tumors.

(A) Hemolymph was collected from Ctrl flies (*EGT/+;+/+*) and Yki flies (*EGT/+; UAS-yki*^{3SA}/+) after four and six days of *yki*^{act} induction. 2-fold serially diluted hemolymph samples were analyzed. Data represent one biological replicate.

bioRxiv preprint doi: https://doi.org/10.1101/2025.10.31.685841; this version posted October 31, 2025. The copyright holder for this preprint (which was not certified by peer review) is the author/funder, who has granted bioRxiv a license to display the preprint in perpetuity. It is made available under aCC-BY 4.0 International license.

Supplementary figure 3. Generation of transgenic fly carrying knock-in of tandem NanoTags on *ImpL2* locus.

(A) Sequence of the synthesized DNA cloned in pUC57 Kan_gw_OK2.

(B) ImpL2-tandem-NanoTag plasmid map.

(C) Sanger sequencing to verify the integration of tandem NanoTags in the C-terminal *ImpL2* locus.

(D and E) Body weights of flies with the denoted genotype. ****$P < 1.0 \times 10^{-4}$ by One-way ANOVA; error bars, SEM; N = 3.

(F and G) Molarity of circulating $\mathrm{ImpL2}^{\mathrm{tNTs}}$ in hemolymph of $ImpL2^{tNTs/+}$ flies measured by sandwich PD-iPCR. X axis indicates the amount of total hemolymph proteins used. error bars, SEM; N = 3.

bioRxiv preprint doi: https://doi.org/10.1101/2025.10.31.685841; this version posted October 31, 2025. The copyright holder for this preprint (which was not certified by peer review) is the author/funder, who has granted bioRxiv a license to display the preprint in perpetuity. It is made available under aCC-BY 4.0 International license.

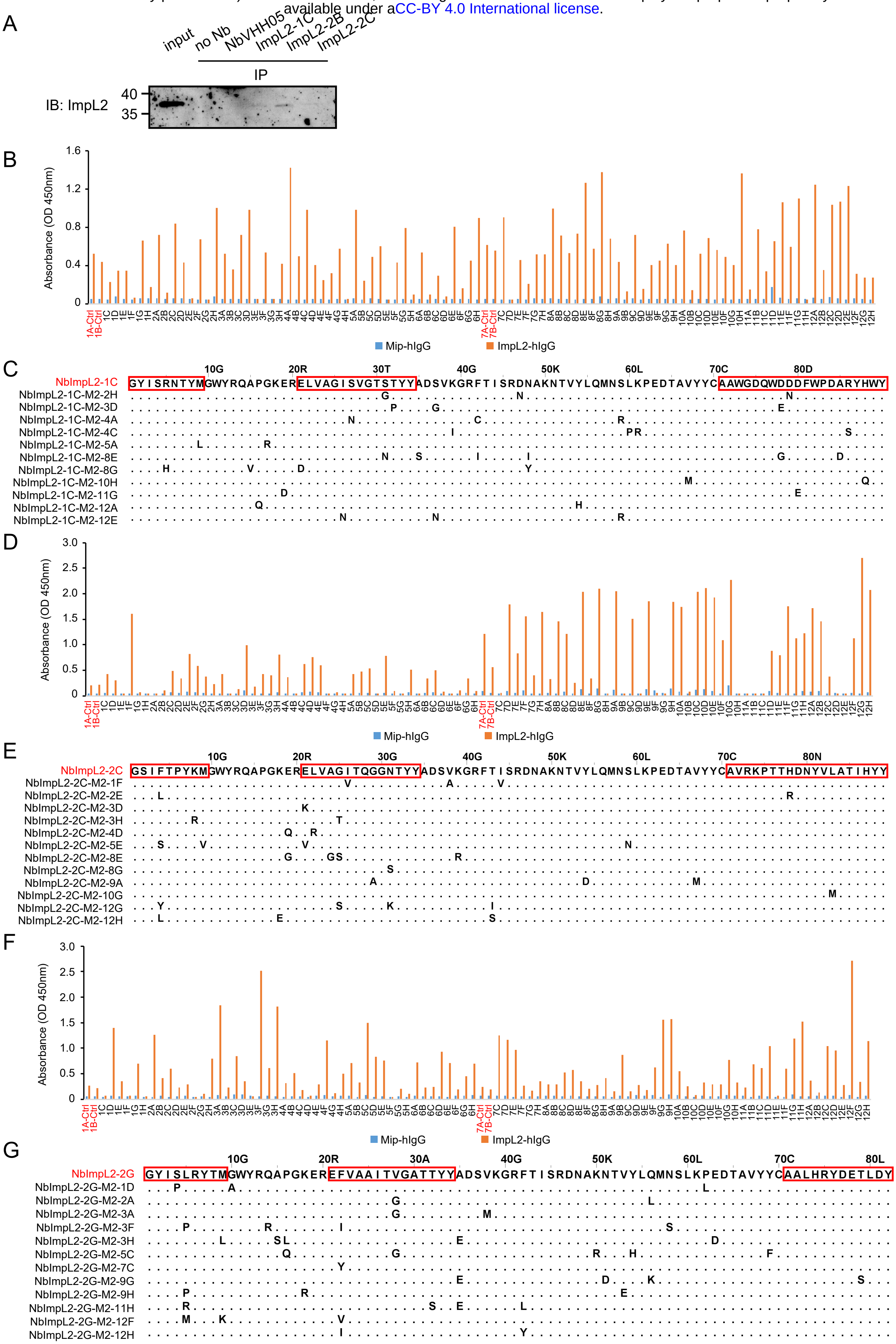

Supplementary figure 1

bioRxiv preprint doi: https://doi.org/10.1101/2025.10.31.685841; this version posted October 31, 2025. The copyright holder for this preprint (which was not certified by peer review) is the author/funder, who has granted bioRxiv a license to display the preprint in perpetuity. It is made available under aCC-BY 4.0 International license.

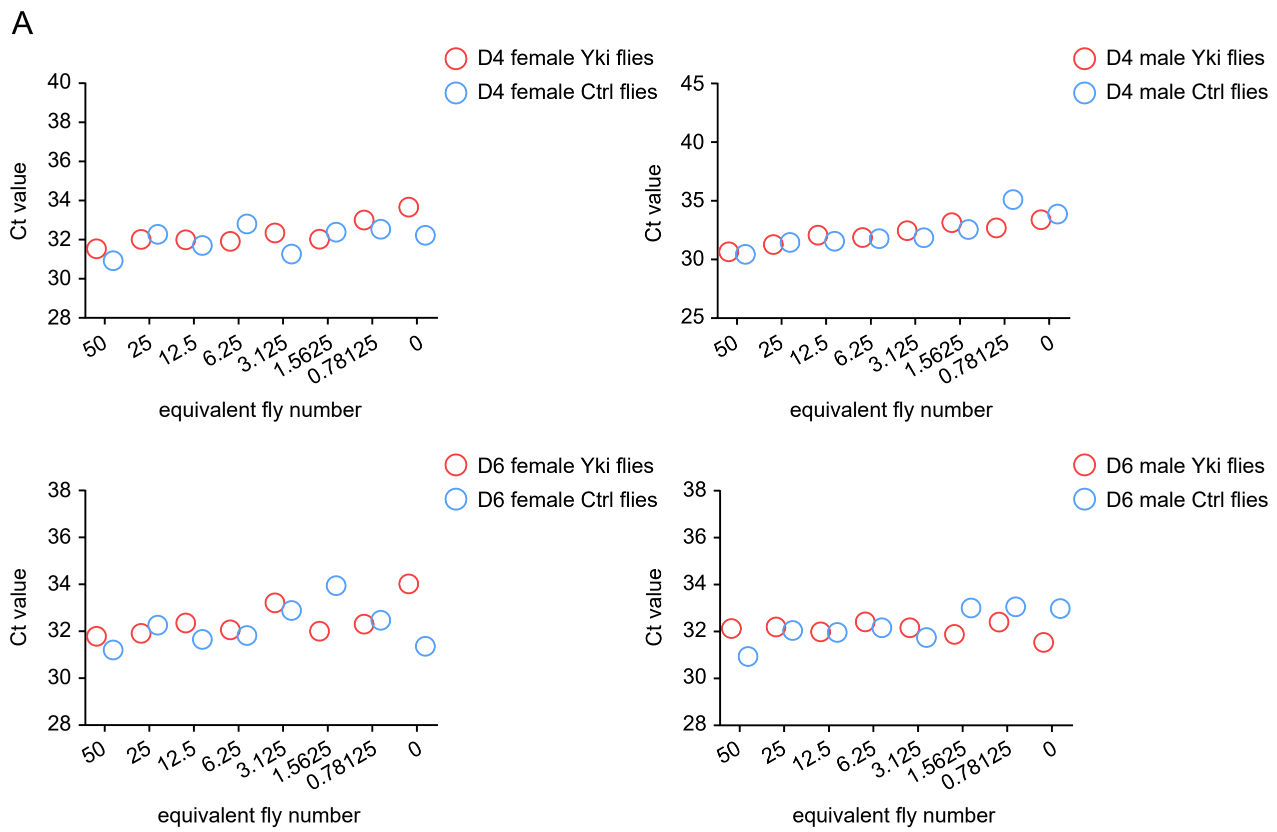

Supplementary figure 2

bioRxiv preprint doi: https://doi.org/10.1101/2025.10.31.685841; this version posted October 31, 2025. The copyright holder for this preprint (which was not certified by peer review) is the author/funder, who has granted bioRxiv a license to display the preprint in perpetuity. It is made available under aCC-BY 4.0 International license.

A

TTAGTCTTCCTCATTCTGCAgttttagagctagaaatagcaagttaaaataaggctagtccgttatcaacttgaaaaagtg
gcaccgagtcggtgcTTTTTTGTAGTACGATCATAACAACGCGgGAGATCAAGTGGGAGGATATGG
GCAACTACAAGTGCATAGCCCGCAACGTCGTCGGAAAGGATACCGCCGATACCTTCGTGT
ATCCCGTACTTgtaagtattcccctacccgccgggttataccaactcatcctaagtaatccctacgtatttttcttgtctttg
cagAATGAGGAAGACGGTAGCGGCTCCTTCGAGGACTTTTGGAAAGGAGAAGATGGTTAG
AGACCATTCGATATAGGTCTCTTTAATAAAGATCCTACCACCAAATAAAAAACAAAAGGCT
ATTGGATTGACGACGGAAATTCATCTAGTTAGTTAGATAGATCAAATGCCTTCGAAGATGC
GTGAAAAGAAATGAAATATGCTTAGAAAAAATACAAAAAAAAAAAAAATCAAAAAAAAAATAT
GATGGATCAAAAGGTCATACGAT

gRNA homology arms linker 127D01-tag BsaI cloning cassette

B

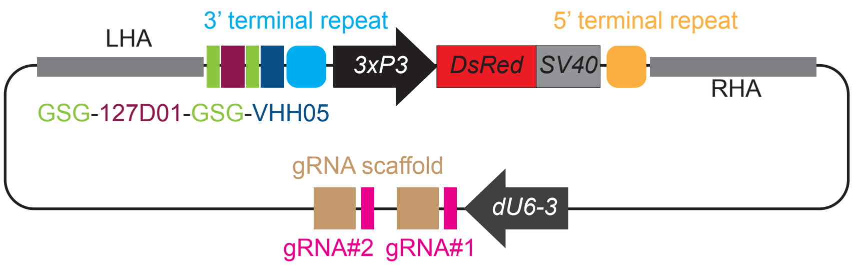

C

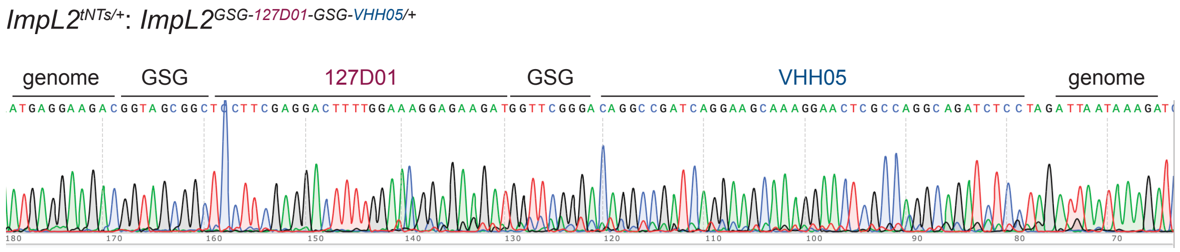

Supplementary figure 3

bioRxiv preprint doi: https://doi.org/10.1101/2025.10.31.685841; this version posted October 31, 2025. The copyright holder for this preprint (which was not certified by peer review) is the author/funder, who has granted bioRxiv a license to display the preprint in perpetuity. It is made available under aCC-BY 4.0 International license.

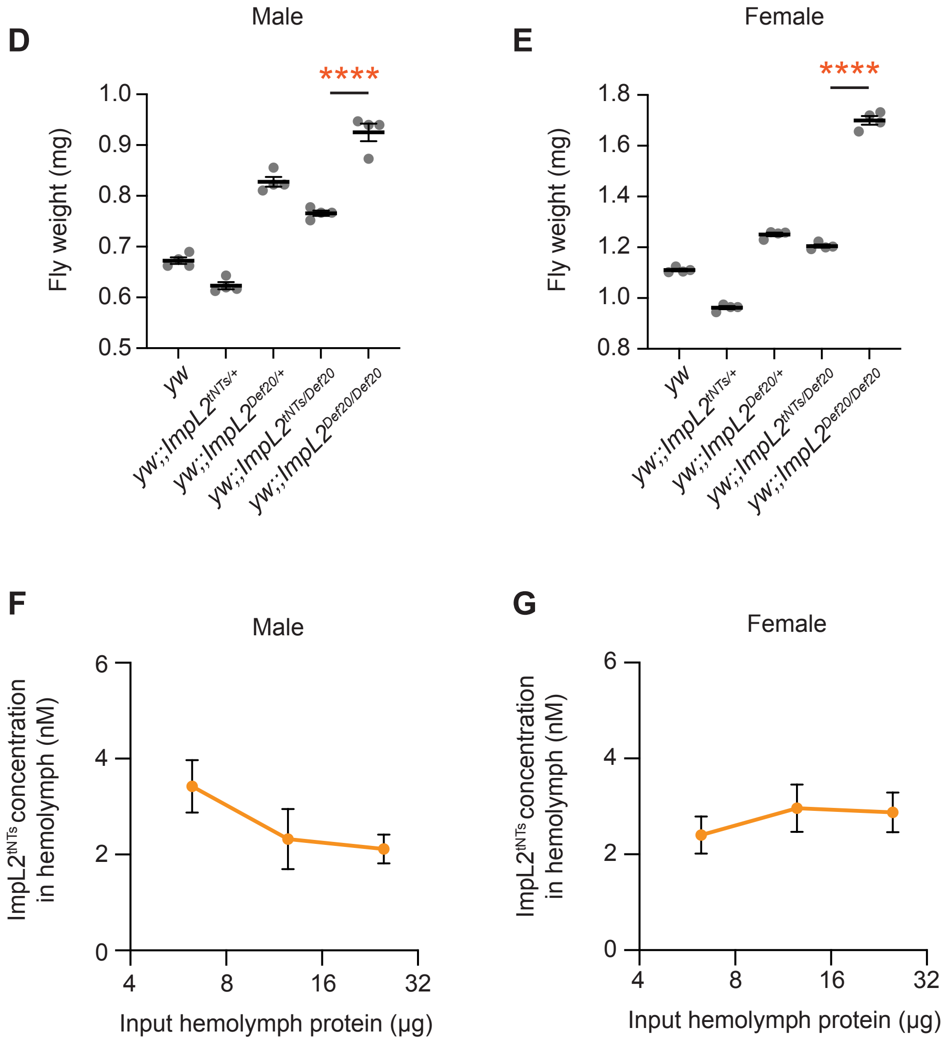

Supplementary figure 3
